# Fiber Optic Localized Surface Plasmon Resonance Sensor Based on Carboxymethylated Dextran Modified Gold Nanoparticles Surface for High Mobility Group Box 1 (HMGB1) Analysis

**DOI:** 10.3390/bios13050522

**Published:** 2023-05-06

**Authors:** Chang-Yue Chiang, Chien-Hsing Chen, Chin-Wei Wu

**Affiliations:** 1 Graduate School of Engineering Science and Technology and Interdisciplinary Program of Engineering, National Yunlin University of Science and Technology, Yunlin 64002, Taiwan; 2 Department of Biomechatronics Engineering, National Pingtung University of Science and Technology, Pingtung 91201, Taiwan; garychc@mail.npust.edu.tw

**Keywords:** high mobility group box 1, carboxymethyl-dextran, gold nanoparticle, localized surface plasmon resonance, biosensor, kinetic binding

## Abstract

Rapid, sensitive, and reliable detection of high mobility group box 1 (HMGB1) is essential for medical and diagnostic applications due to its important role as a biomarker of chronic inflammation. Here, we report a facile method for the detection of HMGB1 using carboxymethyl dextran (CM-dextran) as a bridge molecule modified on the surface of gold nanoparticles combined with a fiber optic localized surface plasmon resonance (FOLSPR) biosensor. Under optimal conditions, the results showed that the FOLSPR sensor detected HMGB1 with a wide linear range (10^−10^ to 10^−6^ g/mL), fast response (less than 10 min), and a low detection limit of 43.4 pg/mL (1.7 pM) and high correlation coefficient values (>0.9928). Furthermore, the accurate quantification and reliable validation of kinetic binding events measured by the currently working biosensors are comparable to surface plasmon resonance sensing systems, providing new insights into direct biomarker detection for clinical applications.

## 1. Introduction

In recent years, clinical studies have pointed out chronic inflammation, such as cancers, diabetes, cardiovascular diseases, allergies, oral diseases, obesity, strokes, and arthritis, will cause mass mortality and significantly increase treatment costs. It has been proven that these chronic diseases are closely related to high mobility group box 1 (HMGB1) [1]. HMGB1 has a molecular weight of 29 kDa and comprises 216 single-chain amino acid polypeptides connected to an acidic C-terminal tail through a short alkaline hinge [2,3]. HMGB1 is a typical damage-associated molecular pattern (DAMP) and a central mediator of lethal inflammation [4,5]. The activated immunocytes or necrotic cells combine with advanced glycation end products (RAGE) and Toll-like receptors upon release from the cells and initiate immunoreaction (inflammation, repair, and recombination) [6,7,8]. Finally, the immune reaction process can induce tissue destruction, fibrosis, necrosis, and death. The general methods to detect HMGB1 include enzyme-linked immunosorbent assay (ELISA) [9,10], Western blot [11], liquid chromatography-mass spectrometry (LC-MS) [12], surface plasmon resonance (SPR) [13,14], electrophoretic mobility shift assay (EMSA) [15], and electrochemical immunosensor [16]. ELISA is the most familiar among these techniques. It is regarded as one of the most convenient, accurate, and reliable methods. However, ELISA requires a long processing time, skilled professionals for the operation, and complex sample preparation. Western blot only performs semiquantitative detection and is time-consuming. LC-MS and SPR provide good quantitative sensitivity, but the instruments are expensive, large, and unlikely to be miniaturized. Therefore, a rapid, accurate, and economical HMGB1 detection method is urgently needed.

The localized surface plasmon resonance (LSPR) biosensor technology is a real-time, rapid response, and high-sensitivity technology [17,18,19]. Gold nanoparticles (AuNPs), as the key components of sensors, have received great attention due to their unique optical and electronic properties and high biocompatibility. They have been widely used to detect biological or chemical molecules, such as protein–protein and protein-nucleic acid binding affinities [18,20,21]. For example, Nath et al. used a label-free optical biosensor to detect streptavidin with a detection limit of 16 nM [22]. Jeon et al. developed a disposable LSPR-based colorimetric sensor for detecting cortisol in the serum [23]. The advantages of LSPR biosensor technology are real time, rapid response, and high sensitivity. However, the interaction between extremely low-concentration samples or low-molecular-weight analytes (e.g., peptides) is still challenging. Due to the refractive index variation induced by molecules being tiny, the difficulty in measurement is increased [24,25,26]. To improve the sensitivity of LSPR biosensors, the previously reported FOLSPR biosensor platform is a promising approach [27,28,29,30,31,32].

In previous studies, mixed self-assembled monolayer (SAM) of 11-mercaptoundecanoic acid (MUA)/6-mercapto-1-hexanol (MCH) bridging molecules were used [33,34]. The MUA is a long-chain molecule with a -COOH functional group. It forms a peptide bond (-CO-NH-) with the amino groups on the surface of biological recognition molecules for a chemical covalent bonding reaction. The MCH has a short chain with a -OH functional group. It is a dilution of thiolate, reducing adhesion and non-specific adsorption between protein molecules [30]. However, the challenge is that the film thickness and spatial structure (steric hindrance) after self-assembly functionalization lead to fewer binding sites for antibody connections and worse anti-adhesion effects [35]. As a result, the detection results are unstable, and the sensitivity is reduced. To overcome these limitations, Carboxymethyl-dextran (CM-dextran) as a binder provides a high surface-to-volume ratio and more -COOH functional groups [36,37]. It increases the conjugate binding sites of biomolecules improving the sensor’s responses [38,39]. CM-dextran is a branched glucose polysaccharide containing the 1,6-α-d-glucopyranosyl bond [40]. It is biocompatible, highly water soluble, highly anti-adhesive, and not toxic. Moreover, it has been widely used in biosensors [41,42,43,44,45].

This study proposes a novel CM-dextran FOLSPR biosensor related to a functionalized CM-dextran-modified binding peptide technique. Chemical covalent bonding is employed in this technique. The experiment used 1-ethyl-3-(3-dimethylaminopropyl)-carbodiimide hydrochloride (EDC) and N-hydroxysuccinimide (NHS) to perform intermolecular cross-linking of CM-dextran and then to modify the biological recognition molecules. The HMGB1 biological standard was tested in the optimal experimental conditions (including modification time, modification concentration, EDC/NHS concentration, antibody modification concentration, and antibody incubation time). The results show that the HMGB1 sensing chip had a wide linear range, high reproducibility, and excellent limit-of-detection (LOD). In addition, compared with the existing MUA/ MCH mixed self-assembled monolayer, the bridge-based HMGB1 molecular detection doubles the detection sensitivity. Notably, this is the first report on detecting HMGB1 in FOLSPR biosensors using CM-dextran as amine coupling reagents to immobilize recognition molecules. This study suggests that the technique can be widely used in fields like biological analysis and detection in food, environment, and clinical medicine.

## 2. Materials and Methods

### 2.1. Materials and Reagents

All chemical reagents are analytical-graded reagents. Hydrogen tetrachloroaurate trihydrate (HAuCl_4_), Phosphate buffered saline (PBS buffer), trisodium citrate solution (C_6_H_5_Na_3_O_7_, ≥99%), (3-Mercaptopropyl)methyldimethoxysilane (MPDMS, >95%), cystamine dihydrochloride (cystamine), dextran 70 (MW ≈ 70,000), sodium periodate (NaIO_4_), 11-mercaptoundecanoic acid (MUA; ≥95%), 6-mercapto-1-hexanol (MCH; ≥97%), 1-ethyl-3-(3-dimethylaminopropyl)-carbodiimide hydrochloride (EDC), N-hydroxysulfosuccinimide (NHS), ethanolamine (EA), mouse IgG, streptavidin, monoclonal anti-HMGB1 antibody, and high mobility group box 1 (HMGB1) protein were from Sigma-Aldrich (St. Louis, MO, USA). Sulfuric acid (H_2_SO_4_; ≥98%), hydrogen peroxide (H_2_O_2_), ethanol, and acetate buffer were purchased from Fluka (Buchs, Switzerland). Ultrapure deionized water (18.2 MΩ·cm^−1^, Milli-Q pure water purification system, Millipore Ltd., Burlington, MA, USA) was used for preparing solutions. The optical fiber probe was multimode plastic-clad silica optical fiber (model F-MBC, Newport), with core and cladding diameters of 400 and 430 μm, respectively, bought from Instant NanoBiosensors Co., Ltd. (Taipei, Taiwan). Sensing chips (poly (methyl methacrylate) (PMMA) plates) were prepared using a CO_2_ laser engraving machine (New Taipei, Taiwan).

### 2.2. Preparation of AuNP Probe

The synthesis of aqueous spherical AuNPs was prepared by the oxidation-reduction method [34]. A 5.2 mL HAuCl_4_·3H_2_O solution at a concentration of 2.43 mM was diluted with 14.8 mL distilled (DI) water to make a 20 mL auric salt aqueous solution. This solution was boiled and slowly mixed with 2.4 mL 1% sodium citrate-reducing solution. The solution slowly turned transparent (Au^2+^) from light yellow (Au^3+^), then into dark black (Au^+^), and claret red AuNPs were formed at last. Afterward, the solution was stirred for 10 min to ensure the reduction process, kept still, and cooled to room temperature. The sol-gel method performed immobilization of AuNP on the optical fiber probe. A 2% MPDMS was prepared in toluene and pre-hydrolyzed for 12 h. The fiber surface of the optical fiber probe was cleaned with soapy water, methanol, and DI water using an ultrasonic bath. The surface was then pickled with piranha solution (H_2_SO_4_ and H_2_O_2_ solution in a volume ratio of 7:3) allowing the surface of the optical fiber probe to carry more -OH functional groups. Afterward, it was cleaned with DI water. The optical fiber probe was immersed in the pre-hydrolyzed 2% MPDMS/toluene solution for 6 h to perform the hydrolysis-condensation reaction of SiO_2_ functional groups. In this order, the optical fiber probe was washed with 1:1 ethanol/toluene solution, ethanol, and DI water and then dried with nitrogen. Afterward, the optical fiber probe was immersed in AuNPs solution for 30 min, and the AuNPs were stably bonded to the surface of the optical fiber probe through S-Au covalent bonding. Finally, the optical fiber probe was washed with DI water to remove the AuNPs not bonded on the optical fiber probe. After nitrogen drying, the UV-Visible/NIR Spectrophotometer (Hitachi UH5700, Tokyo, Japan), transmission electron microscopy (TEM, JEM-2100Plus, Tokyo, Japan), and ultra high-resolution thermal field emission scanning electron microscope (FESEM, JEOL JSM-7610FPlus, Tokyo, Japan) were used to measure the absorption spectra of the synthetic AuNPs and the AuNPs on the surface of the immobilized optical fiber probe. The spectral characteristic peak absorption wavelength position and absorption strength of the spherical AuNPs solution were identified to verify the shape and size. The test was repeated three times for each data point, and the data were represented by an average value and standard deviation (mean ± SD).

### 2.3. Microfluidic Sensing Chip and FOLSPR Sensing System

**Figure 1:**
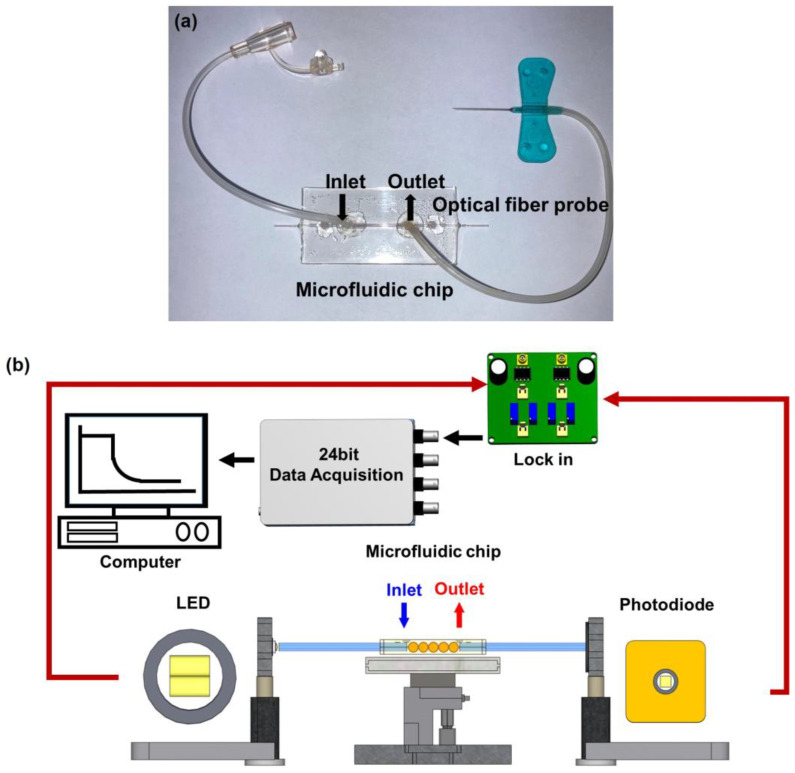
FOLSPR sensor. (**a**) Microfluidic sensing chip, and (**b**) schematic representation of the experimental setup of the sensing system.

Microfluidic sensing chip was composed of a PMMA substrate. It comprises a cover and bottom plates. The dimensions of each cover plate are 25 mm (width) × 50 mm (length) × 2 mm (thickness). The actual surface of AuNP coated zone is 25.1 mm^2^ (0.4 mm (the core diameter) × π × 20 mm (length)). The bottom plate involved a microfluidic technique, and the sample micro-channel is 800 μm (depth) × 800 μm (width) to accommodate a 730 μm optical fiber probe with a volume of 35 μL. A CO_2_ laser engraving machine processed the cover plate. A micro-channel access hole of about 0.2 cm diameter could be connected to the plastic flow pipe to import and export fluids. The sensing chip was packaged with 3M double-sided adhesive to bond the cover plate and bottom plate. Afterward, DI water was injected into the sensing area for cleaning. Finally, a complete microfluidic sensing chip was obtained, as shown in Figure 1a.

Figure 1b is the schematic diagram of a FOLSPR biosensor system. The sensing system used a green LED with a microlens (model IF-E93, Industrial Fiber Optic, Inc., Tempe, AZ, USA) as the light source, with a peak wavelength of 530 nm and a circuit system (including 1 kHz square wave signal and signal amplifier). One end of the light source was coupled to the inside of the optical fiber sensing chip for multiple total internal reflections. This helps generate evanescent waves on the surface of the sensing area. These evanescent waves excite AuNPs to generate the LSPR effect. Meanwhile, the other end was connected to the light exit of photodetection. The experiment used a high-stability photodiode (S1336-18BK, Hamamatsu, Tokyo, Japan) to detect the light intensity. The circuit system was linked with a data acquisition card lock-in module (dynamic signal acquisition module USB-9234 and Lab-VIEW 2019 software, National Instrument, Austin, TX, USA) to transform the light into voltage signals (real-time light intensity). The generated voltage signals were captured and analyzed by a computer to transform the light into voltage signals (real-time light intensity). The generated voltage signals were captured and analyzed by a computer.

### 2.4. Preparation of CM-Dextran Solution and Functionalization of CM-Dextran Sensing Chips

**Figure 2:**
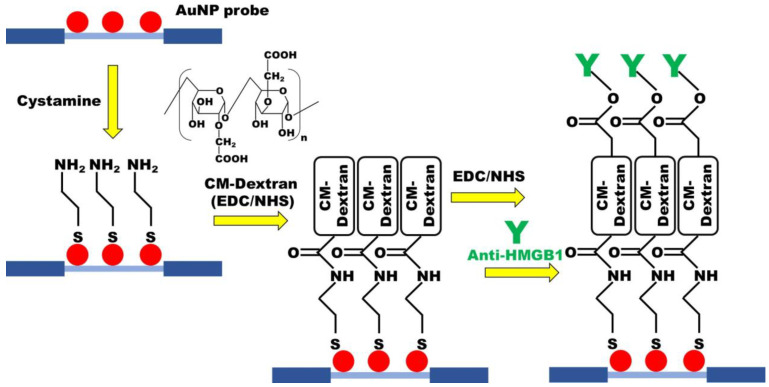
The CM-dextran-based FOLSPR chip experimental conditions.

Carboxymethyl-dextran (CM-dextran) was synthesized using the previously reported protocol with minor modification [46,47]. Briefly, CM-dextran was formed by dissolving 3 g of dextran in 10 mL of NaOH 100 mM, adding 1 M solution of bromoacetic acid, and then shaking at room temperature in the dark for 16 h. The functionalization of the sensing chip was performed by chemical covalent bonding (Figure 2). To form abundant amine (-NH _2_) groups on the AuNP surface of the optical fiber, which was modified with cystamine. CM-dextran is then dissolved in phosphate-buffered saline (PBS, pH = 7.4) and mixed with EDC and NHS for activation, allowing cystamine to react with the carboxymethyl groups on CM-dextran. Subsequently, a second activation step using EDC and NHS was used for intermolecular cross-linking of CM-dextran, forming peptide bonds (-CO-NH-) with amino groups of the antibody to complete the antibody fixed. Residual active groups were saturated by injection of ethanolamine. UV/Vis-NIR spectrum and Energy-dispersive X-ray spectroscopy (EDS) were used to identify material analysis in the experimental process.

The optimum conditions for the modification of the sensing chip were discussed in the experiment. First, the modified AuNPs optical fiber was used for sensing chip packaging. Then, 35 μL 0.02 M cystamine solution was injected into the sensing chip for 30 min to form AuNP-cystamine. Afterward, DI water was applied to remove uncombined cystamine. The CM-dextran (1%, 2.5%, 5%, 7.5%, and 10%)/EDC (0.2 M)/NHS (0.05 M) mixed solutions were injected on the surface of AuNP-cystamine, with a CM-dextran immobilization time of 0.5, 1, 1.5, 2, and 2.5 h. The AuNP-cystamine- CM-dextran was formed on the surface. It was removed by using ionized water. The -COOH functional group on the surface of CM-dextran was activated using the mixed liquor of EDC (0.2 M) and NHS (0.05 M) for 20 min. A 50 μL anti-HMGB1 (50, 75, 100, 125, and 150 μg/mL) was applied. The incubation time was 0.5, 1, 1.5, and 2 h for AuNP-cystamine-CM dextran-anti-HMGB1 functionalization. The uncombined anti-HMGB1 was removed by PBS buffer. Finally, 1 M ethanolamine (pH 8.5) aqueous solution was used for 7.5 min to inactivate the unreacted -COO- and prevent nonspecificity. Inject PBS solution into the sensor chip for verification after step-by-step modification. The gradual modification process of the sensing chip experiment was verified by UV/Vis-NIR spectrum. The FOLSPR system was used for real-time signal measurement.

### 2.5. Preparation of Functional MUA/MCH Sensing Chip

**Figure 3:**
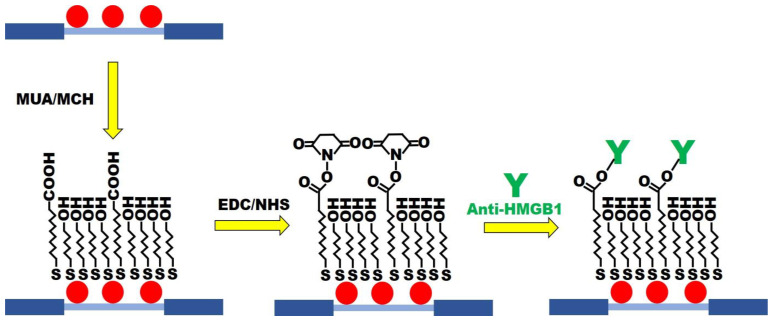
The MUA/MCH-based FOLSPR chip experimental conditions.

MUA/MCH sensing chips were functionalized (Figure 3) in accordance with the previous procedure with slight modification [30]. The optical fiber of modified AuNPs was immersed in the ethanol solution with MUA (2 mM) and MCH (2 mM) in a mole ratio of 1:4. It was kept still at room temperature for 12 h to form the mixed SAM. It was then washed with ethanol and dried with N_2_. Afterward, the AuNPs-MUA/MCH optical fiber was used for sensing chip packaging. A 35 μL aqueous solution of EDC (0.2 M) and NHS (0.05 M) was injected into the sensing chip to activate the -COOH group for 30 min. Then, the DI water was injected for washing. A 35 μL anti-HMGB1 (100 μg/mL) solution was injected for a 1 h incubation reaction. The PBS buffer was injected into the solution to wash out the uncombined anti-HMGB1. To inactivate the unreacted -COO-, 1 M aqueous ethanolamine solution with pH 8.5 (7.5 min) was used for washing. Finally, the PBS solution was injected to wash the immobilized antibody’s surface. The FOLSPR system was used for real-time signal measurement in the gradual modification process.

### 2.6. Sample Preparation

The stock HMGB1 standard solution was diluted with PBS and stored in a refrigerator at −20 °C. The sample was prepared and used in the same week to avoid the inactivated HMGB1-inducing errors. The concentration range of the prepared HMGB1 standard solution was 1 × 10^−10^ to 1 × 10^–6^ g/mL, which was stored at 4 °C for further use. The PBS buffer of pH 7.4 was injected into the sensing chip as the baseline. The prepared HMGB1 standard solutions (1 × 10^−10^, 1 × 10^−9^, 1 × 10^−8^, 1 × 10^−7^, 1 × 10^−6^ g/mL) were tested to obtain corresponding signal responses. Each was tested by three different sensing chips, and the data were shown by average values and standard deviations (average value ± standard deviation). A linear relationship between signal responses and concentrations was drawn to obtain the calibration curve. Finally, Origin 2021 (OriginLab, Northampton, MA, USA) was used for statistical analyses of data.

## 3. Results

### 3.1. Material Analysis

**Figure 4:**
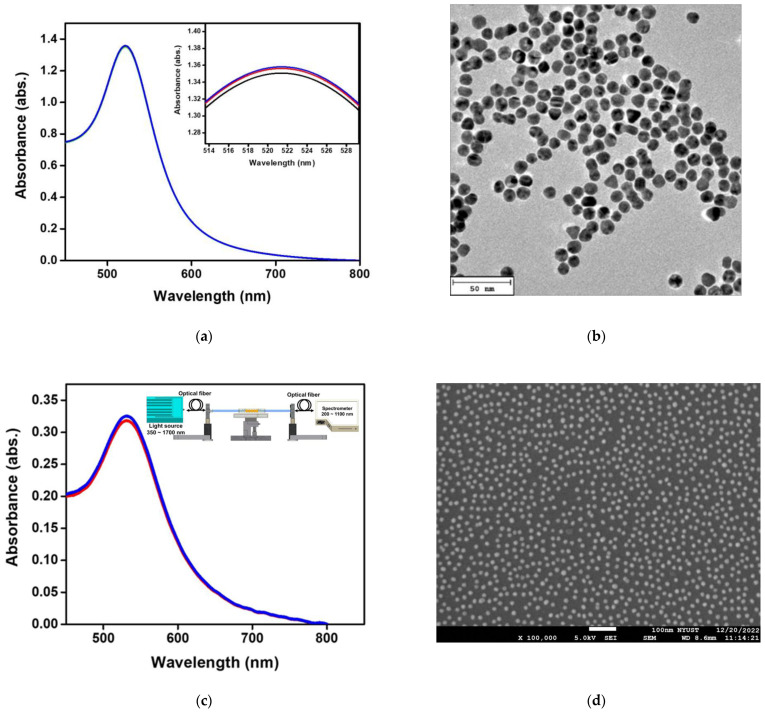
Structural characterizations of materials. (**a**) Absorption spectra of Au nanoparticles (AuNPs) in the aqueous medium in the visible region; (**b**) TEM image of AuNPs; (**c**) a fiber optic spectrometer was used to measure the absorption spectrum of AuNPs on the surface of the probe in the visible region in the aqueous medium; (**d**) FESEM image of AuNPs on the fiber core surface. The test was repeated three times for each data point (*n* = 3), and the data were represented by an average value and standard deviation (average value ± standard deviation).

Figure 4a shows the absorption spectrum of synthetic aqueous spherical AuNP solution in a UV-Visible/NIR spectrophotometer. The maximum value of absorption peak occurs at 521 ± 0.6 nm. Figure 4b shows the TEM image of the aqueous spherical AuNP solution. The AuNPs were complete spheres with no aggregation and a mean particle size of 12.2 ± 0.6 nm. Figure 4c shows the extinction spectrum of the fiber surface modified AuNPs measured by the self-mounted fiber-optic spectral system as shown in Figure 4c insert, including the spectrometer (Ocean Optics, QEPro, optical resolution: 1.2 nm), a white light source of the 350 nm ~ 1700 nm (OTO Photonics, LS-HA) and two optical fiber of the core is 400 μm (Ocean Optics, numerical aperture: 0.22) [48]. The absorption peak of AuNPs occurred at 532 ± 0.8 nm. Figure 4d shows AuNPs on the sensing chip fiber surface measured by ultra-high resolution thermal FESEM. It was obvious that the nanoparticles were spherically dispersed on the fiber surface without aggregation, and FESEM calculated the AuNPs particle size. The results show that the mean particle size is 13.4 ± 1.2 nm (200 particles).

**Figure 5:**
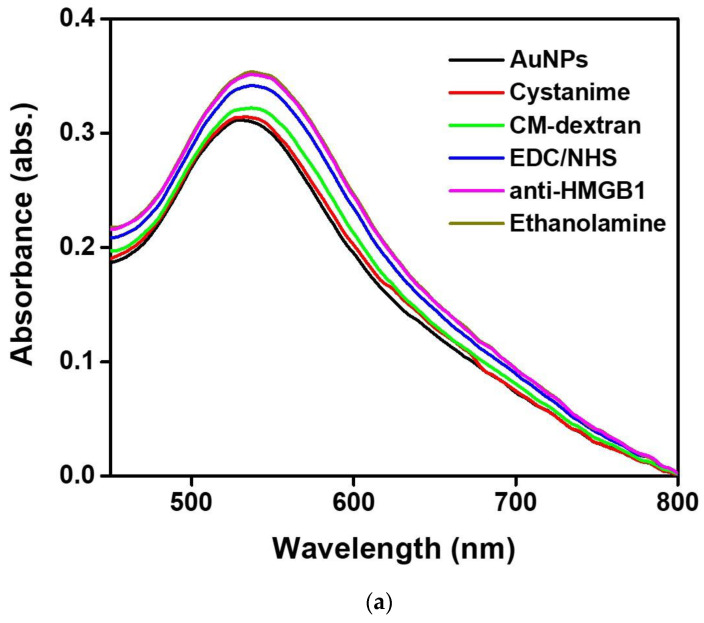

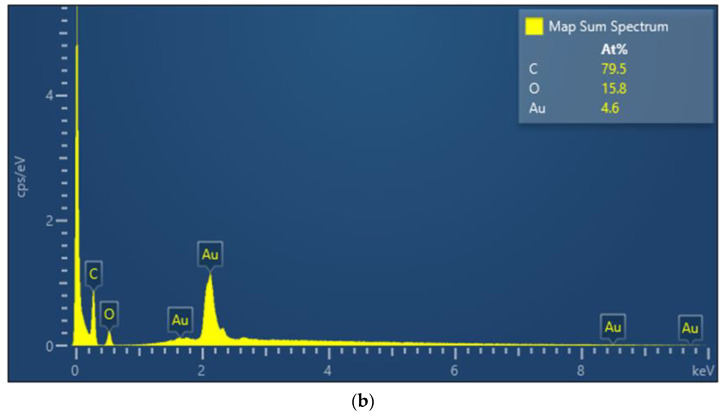
(**a**) Gradual functionalization of AuNP, cystamine, CM-dextran/EDC/NHS, EDC/NHS, anti-HMGB1, and ethanolamine in the probe modification spectrogram (measured in the PBS buffer); (**b**) EDS measurement verification.

In this study, we use UV-Vis spectroscopy to monitor the molecular step-by-step modification process of a sensor chip experiment. Figure 5a shows the gradual functionalization process of AuNP, cystamine, CM-dextran/EDC/NHS, EDC/NHS, anti-HMGB1, and ethanolamine in the optical fiber sensing area measured by a fiber optic spectrometer (Measured in the PBS buffer). The figure shows that the characteristic peak and wavelength of AuNPs on the optical fiber surface sensing area were at 531.1 nm at the beginning (absorbance was 0.3125). After gradual modification, it was observed that the last characteristic peak of AuNP-cystamine-CM-dextran/EDC/NHS-EDC/NHS-anti-HMGB1-ethanolamine was at 537.6 nm (absorbance was 0.35886), showing a redshift (displacement was 8 nm). The results show the variation of the local refractive index near the AuNPs surface and the electron loss on the AuNPs surface. It indirectly proves the immobilization of molecules on the surface of AuNPs or the conjugation of recognition molecules analyte. In addition, the absorbance peak increased from 0.3125 to 0.35886. This is consistent with the observation of the absorption increase and spectral redshift after combining chemical and biological molecules proved in prior studies [49,50,51]. Therefore, these results prove the successful functionalization of the sensing probe modified by AuNP-cystamine-CM-dextran-EDC/NHS-EDC/NHS-anti-HMGB1-ethanolamine. Figure 5b shows the elemental analysis of AuNP-cystamine-CM dextran surface in the optical fiber sensing area measured by Energy-dispersive X-ray spectroscopy (EDS). The C, O, and Au signals from the functionalization of AuNP-cystamine-CM dextran were detected, meaning that the components were CM dextran and Au.

### 3.2. Principle of FOLSPR Sensors

It is well-known that the physical characteristics of LSPR generated on the surface of AuNPs are susceptible to the external refractive index [17,52,53,54]. Especially during biological detections, the density of recognition molecules and molecular weight will lead to the sensitivity of the biomolecular binding [55,56]. This study is the first to use CM-dextran as bridging molecules to increase the density of recognition molecules and improve the sensitivity of FOLSPR sensing technology. The principle of FOLSPR is that after the light is led in the optical fiber. After multiple total internal reflections in the optical fiber core, the evanescent wave energy is generated on the surface of the cladding-off cylindrical fiber. When the surface AuNPs absorb the evanescent wave energy, the electrons on the surface of AuNPs generate the LSPR effect [57,58]. The intensity of this resonance will vary with the number of modification molecules on the surface of AuNPs, resulting in differences in light intensity. The difference in light intensity will increase the absorbance or change the refractive index of the external environment in the bonding process of the recognition molecules (e.g., capture antibodies, DNA, RNA, primers, or aptamers) and analytes. Therefore, the light intensity change can be recorded instantly as the biosensor analysis tool [48]. In this study, light signal I_0_ is the light intensity response in the blank solution under a PBS buffer. I_A_ is the light intensity response under the analyte solution. The sensor response is defined as (I_0_ − I_A_)/I_0_ = ΔI/I_0_ and is implemented by comparing the collected light intensity from a fiber probe immersed in a sample solution (I_A_) containing the target protein at defined concentrations to that of immersed in a blank solution (I_0_), i.e., the normalized light intensity change. The light’s real-time signal response intensity declines as the analyte concentration increases. The quantitative testing analysis was obtained by concentration and signal responses.

**Figure 6:**
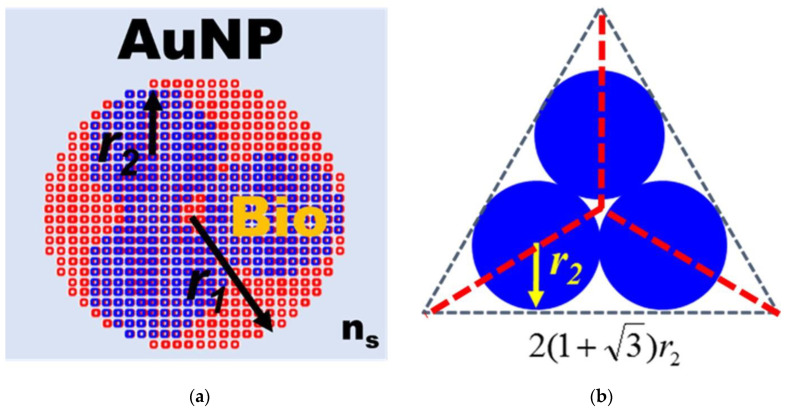
Structural diagram of AuNP surface modification biomolecules simulated by DDA. (**a**) Structure of AuNP surface modification biomolecules; (**b**) Biomolecular structure.

**Figure 7:**
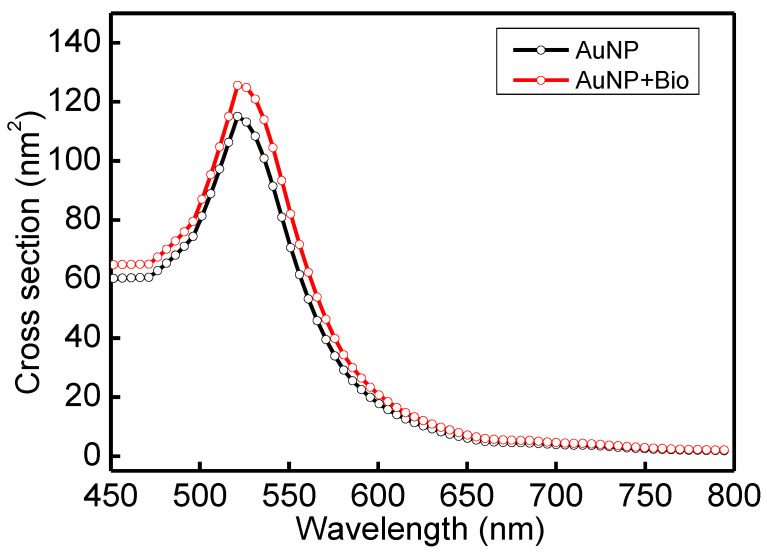
Extinction spectrum of AuNP surface modification biomolecules simulated by DDA.

In order to understand the optical properties of biomolecules interacting with nanoparticles, this study used discrete dipole approximation (DDA) to simplify that model and simulate nanoparticle surface modification biomolecules’ extinction spectrum [59]. It can be used to design a real-time light intensity detection system. The simulation structure and parameters are shown in Figure 6a and Table 1. The simulation wavelength range is 450–800 nm. The simulation structure contained a circular AuNP with a size of 13 nm (reference Figure 4b TEM image). The refractive index (N_metal_ = *n* + i*k*) had real part *n* and imaginary part *k* [60]. Biomolecules were adsorbed on the circular AuNP surface. Biomolecules consist of three spheres with a radius and refractive index of 3.2 nm and 1.45, respectively. Their size is about a 13 nm Y-shaped structure, as shown in Figure 6b. The external environment was a phosphate-buffered saline (PBS buffer) with a refractive index of 1.42. The results of the extinction spectrum of nanoparticle surface modification biomolecules simulated by DDA are shown in Figure 7. The LSPR peak wavelength changes were not apparent before and after the AuNP surface modification of biomolecules. Additionally, the extinction cross-section increased from 108.3 nm^2^ to 120.9 nm^2^ at the wavelength of 530 nm with a variable rate of about 11.1%. It shows that it is easier to observe the change of light intensity as the indicator of biosensor analysis than the wavelength shift. Based on the experimental results, the before (at 530 nm, Figure 5 black line AuNPs) and after (Figure 5 purple line anti-HMGB1) absorbance was 0.31028 and 0.35317, respectively. The AuNPs surface was modified with biomolecules. With an increased absorbance, the light intensity decreased after the AuNPs surface was modified with biomolecules. The light intensity variation rate was 9.4% (10^−0.31028^ − 10^−0.35317^/10^−0.31028^), meaning the biomolecules modified the AuNPs surface. This finding matched the trend of simulation results. It is also the reference for choosing the wavelength of 530 nm as the light source for the FOLSPR biosensor system in this study.

### 3.3. Optimization of the Sensor

**Figure 8:**
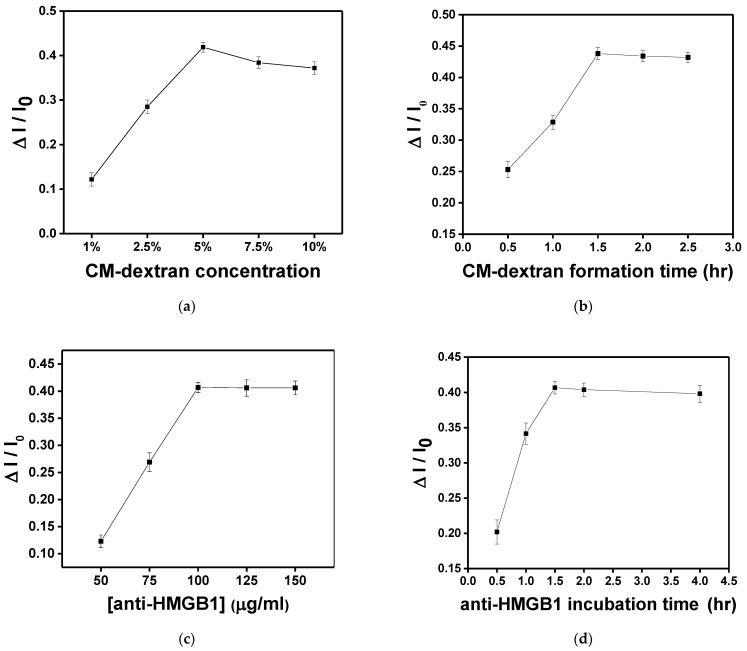
In each experiment, 10^−8^ g/mL HMGB1 was injected for 15 min of molecular binding to obtain the signal response. Each point is the mean of three repeated measurements. (**a**) Effect of CM-dextran concentration ratio (1%, 2.5%, 5%, 7.5%, and 10%) in the starting solution on the sensor response. (**b**) Effect of immersion time (0.5, 1, 1.5, 2, and 2.5 h) given to form CM-dextran on the sensor response. (**c**) Effect of concentration of anti-HMGB1 (50, 75, 100, 125, and 150 μg/mL) used for the bioconjugation process on the sensor response. (**d**) Effect of incubation time (0.5, 1, 1.5, 2, and 4 h) of anti-HMGB1 used for bioconjugation on the sensor response.

In this study, the sensing chip formed surface functionalization through chemical bonding reactions. In each experiment, 10^−8^ g/mL HMGB1 was injected for 15 min of molecular binding to obtain the signal response. First, the AuNP-cystamine surface was modified individually with 1%, 2.5%, 5%, 7.5%, and 10% (weight%) CM-dextran solution. As shown in Figure 8a, the increase is apparent when the concentration was 1%, 2.5%, and 5%. When the concentration was 5%, the response of HMGB1 was the largest (ΔI/I_0_ = 0.419 ± 0.011). Signals gradually decreased after 7.5% and 10%. This is because the viscous CM-dextran solution resulted in steric hindrance. Therefore, the concentration of CM-dextran was set at 5% for subsequent experiments. The CM-dextran immobilization time was 0.5, 1, 1.5, 2, and 2.5 h. It was observed that the immobilization gradually reached saturation in 1.5 h (Figure 8b). Therefore, the subsequent work used 1.5 h as the CM-dextran formation time. The AuNP-cystamine-CM-dextran was formed on the surface of the probe, and the unbonded CM-dextran was removed by ionized water. Afterward, the -COOH functional group on the surface of CM-dextran was activated using the mixed liquor of EDC (0.2 M) and NHS (0.05 M) for an amine coupling reaction with activation times of 20 min. At this time, 50 μL anti-HMGB1 (50, 75, 100, 125, and 150 μg/mL) was injected to influence the signal response by antibody modification. It was observed that when the concentration of anti-HMGB1 was 100 μg/mL, the signal response was the highest (Figure 8c). Then, the signal response became smooth. Therefore, the concentration of anti-HMGB1 was set as 100 μg/mL for subsequent experiments. The incubation time of anti-HMGB1 (0.5, 1, 1.5, 2, and 4 h) was closely related to the stability of the chemical covalence of recognition molecules. Different from the traditional monolayer, CM-dextran had multiple binding points. It was observed that the signal response exhibited the maximum signal decline at 1.5 h and then presented a smooth trend (Figure 8d). Therefore, the anti-HMGB1 incubation time was 1.5 h in subsequent experiments.

### 3.4. Comparison of CM-Dextran and MUA/MCH Sensing Chip Modification Anti-HMGB1 Responses

**Figure 9:**
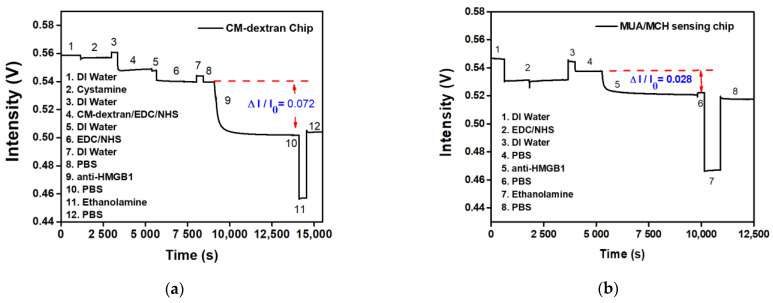
The signal responses of anti-HMGB1 modified by different bridging molecules were compared; (**a**) a CM-dextran sensing chip; (**b**) and an MUA/MCH sensing chip.

The signal responses of anti-HMGB1 modified by different bridging molecules were compared. The experiment used two surface modification methods: (a) fixing by CM-dextran, and (b) fixing by MUA/MCH. Afterward, the -COOH on CM-dextran or MUA was activated by EDC/NHS, which bonded with amines on anti-HMGB1 to modify the surface of the optical fiber probe. A PBS buffer washed out the uncombined anti-HMGB1. To inactivate the unreacted -COO-, 1 M aqueous ethanolamine solution of pH 8.5 was used for washing. The signal response at each stage was evident in the real-time signal graph. It indirectly proves the steps of antibody immobilization on the AuNP surface or the antibody-analyte interactions. The difference in signal responses between the two sensing chips when anti-HMGB1 was applied could be seen from the real-time signal change in Figure 8a,b. The CM-dextran sensing chip developed in this study had relatively abundant surface bonding points. The molecular bonding of the carboxyl group on anti-HMGB1 and CM-dextran was relatively reduced in the overall signal (ΔI/I_0_) to 0.072 (Figure 9a). However, the MUA/MCH sensing chip was a mixed monolayer, and the molecules were arranged mainly by the Van der Waals force. The steric hindrance was formed in the arrangement process, leading to fewer bonding points. It was observed that the overall relative signal reduction (ΔI/I_0_) is 0.028 when modifying anti-HMGB1 (Figure 9b). This indicates that the relative signalization of the CM-dextran sensing chip was 2.57 times that of the MUA/MCH sensing chip with modifying the recognition molecules.

### 3.5. Nonspecific Adsorption Test

**Figure 10:**
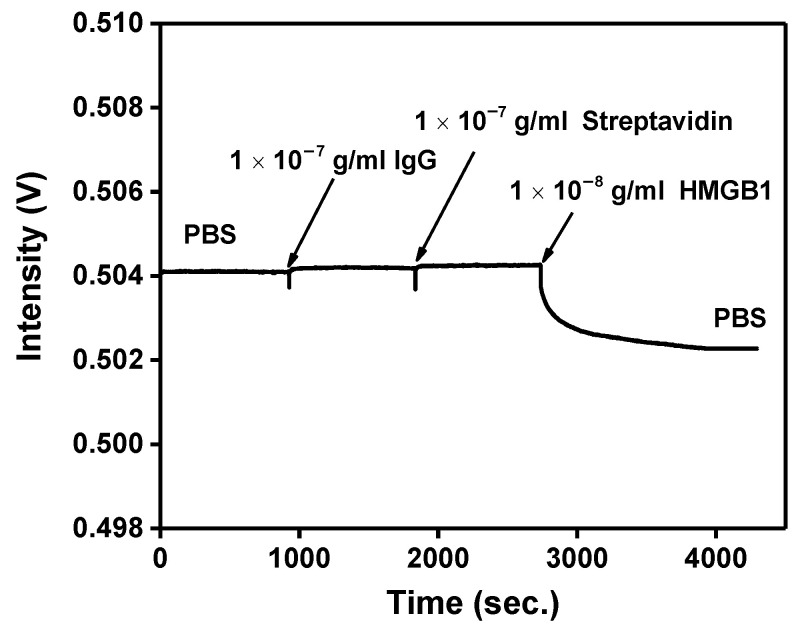
Nonspecific adsorption and specificity tests. The signal response of HMGB1 antibody-functionalized FOPPR sensor in response to IgG (1.0 × 10^−10^ g/mL), Streptavidin (1.0 × 10^−7^ g/mL), and HMGB1 (1.0 × 10^−8^ g/mL) solutions.

The probe experiment has been determined, and the nonspecific adsorption problem will finally be solved. Nonspecific adsorption is a universal problem in biosensor research. The hydroxyl (-OH), methyl (-CH_3_), or ethanolamine (pH 8.5) are generally used to inactivate and block excessive NHS ester to avoid or reduce nonspecific adsorption phenomenon. Prior studies have proven that ethanolamine (pH 8.5) can block excess binding sites and reduce the nonspecific adsorption problem. First, the FOLSPR system monitored the recognition molecule-analyte binding reaction in the buffer solution. According to the real-time signal response in Figure 10, there was no signal change after injection of 10^−7^ g/mL IgG and Streptavidin. These results show that nonspecific binding on the CM-dextran sensing chip’s surface seemed negligible. The ethanolamine could prevent interference effectively. The signal drop was generated when 10^−8^ g/mL HMGB1 was injected, confirming that the specific bonding interaction between the immobilized anti-HMGB1 on the AuNP surface and the HMGB1 in solution induced the measured signal change.

### 3.6. Sensitivity of the Biosensor

**Figure 11:**
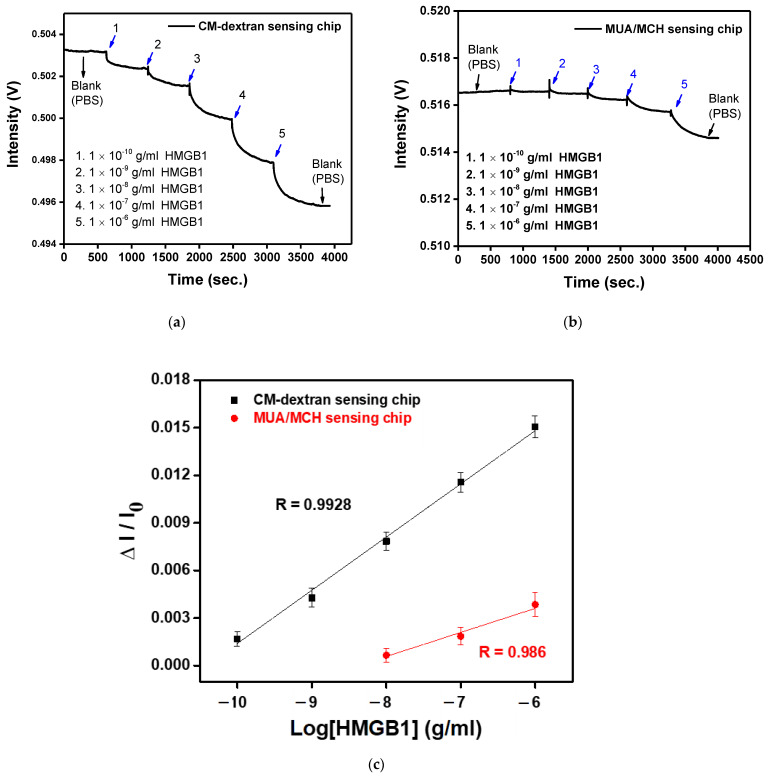
(**a**) The anti-HMGB1 functionalized CM-dextran sensing chip signal with the injection of different HMGB1 concentrations of (1) 1.0 × 10^−10^, (2) 1.0 × 10^−9^, (3) 1.0 × 10^−8^, (4) 1.0 × 10^−7^, and (5) 1.0 × 10^−6^ g/mL. (**b**) The anti-HMGB1-functionalized MUA/MCH sensing chip with the injection of different HMGB1 concentrations of (1) 1.0 × 10^−10^, (2) 1.0 × 10^−9^, (3) 1.0 × 10^−8^, (4) 1.0 × 10^−7^, and (5) 1.0 × 10^−6^ g/mL. (**c**) Calibration curve for HMGB1 by CM-dextran and MUA/MCH sensing chip in FOLSPR biosensor. Each point is the mean of three repeated measurements.

The main purpose of this study is to design a rapid, simple, and sensitive real-time detection technology for HMGB1 molecules. Here, the FOLSPR system successfully demonstrated high HMGB1 molecular binding kinetics sensitivity and used CM-dextran as bridging molecules for low-cost detections. The experimental research performed HMGB1 standard solution detection (concentration range was 1 × 10^−10^ to 1 × 10^−6^ g/mL) on the FOLSPR sensing chip functionalized by two bridging molecules. First, the PBS solution was injected as the baseline (600 s). Then, the final measured 300 s signals were averaged as I_0_, and multi-concentration measurements were performed (each concentration was measured for 600 s). The real-time signal response between the recognition molecules and the analyte is shown in Figure 11a,b. It increased with each analyte concentration (the time to reach 90% of the equilibrium signal level) for about 400 s, and steady signals could be observed within 10 min. The concentration and relative signal quantity were plotted. The performance of the two kinds of sensing chips was linear for all concentrations (Figure 11c). The relative linearity (R^2^) was 0.9928 (CM-dextran sensing chip) and 0.986 (MUA/MCH sensing chip), respectively. The standard correction curve was used to estimate the limit of detection (LOD). The CM-dextran sensing chip detection HMGB1 was 43.4 pg/mL (1.7 pM), and the LOD of MUA/MCH sensing chip detection HMGB1 was 3970 pg/mL (158.8 pM). The detected concentration range was enhanced by two orders of magnitude. In the past, Vilma et al. [10] employed ELISA to detect HMGB1 at a LOD of 0.4 ng/mL. Wang et al. [11] used Western blotting to detect HMGB1 at a LOD of 1 ng/mL. Other HMGB1 detection methods include an SPR with a LOD of 900 ng/mL [13] and an electrochemical immunosensor with a LOD of 2 ng/mL [61]. It was apparent that the CM-dextran FOLSPR biosensor had excellent detection sensitivity. This shows that the CM-dextran proposed in this research offered better performance in immune response analysis. In addition, FOPPR sensing methods offer more straightforward procedures, point-of-care detection (which can explore kinetic estimation between capture molecules and analytes), and relatively short analysis times compared to traditional detection methods such as ELISA or Western blotting. Finally, the molecular binding kinetics was estimated to determine the association rate constant (ka) and dissociation rate constant (kd) for anti-HMGB1 on the AuNP surface and HMGB1 in PBS; the values of these constants are 2.66 ± 0.2 × 10 ^5^ M ^−1^ s ^−1^ and 5.71 ± 0.8 × 10 ^−2^ s ^−1^, respectively. Afterward, the affinity constant Kf (Kf = ka/kd) was calculated using ka and kd values, i.e., 4.66 ± 0.59 × 10 ^6^ M ^−1^ (number of samples = 3). It coincides with the constant rate result estimated using a surface plasmon resonance sensor (Biocore T200, GE Healthcare, Anaheim, CA, USA) (ka = 2.86 ± 0.03 × 10 ^5^ M ^−1^ s ^−1^, kd = 0.119 ± 0.00 s ^−1^ and Kf = 2.40 ± 0.03 × 10 ^6^ M ^−1^) in the literature. The results show that our method has simple operation, quick response, real-time detection, low sample consumption (0.35 mL), good analytical performance, and avoids using enzymes. Hence, it has good prospects in other areas in the future.

### 3.7. Sensing Chip Repeatability and Stability Tests

**Figure 12:**
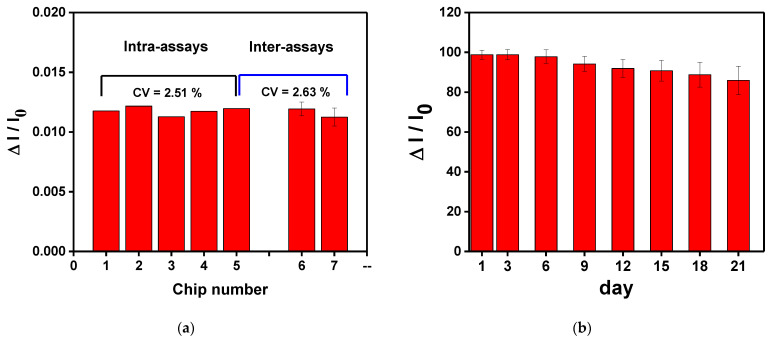
(**a**)The intra-assay and inter-assay reproducibility values of the CM-dextran biosensor chips were checked by repeated measurements of the same concentration of HMGB1. (**b**) Storage stability of the AuNP-CM-dextran-anti-HMGB1 probe onto sensing chip. Each point is the mean of five repeated measurements.

To study the repeatability and stability of the CM-dextran sensing chip, the chip was measured three times in the optimum conditions. First, the intra-batch and inter-batch repeatability values of the CM-dextran biosensor chip were checked by repeatedly measuring the HMGB1 at the same concentration. As shown in Figure 12a, the RSD values of relative signal (ΔI/I_0_) of 1.0 × 10^−8^ g/mL HMGB1 in the intra-batch and inter-batch tests are 2.51% and 2.63%, respectively. These results show that the assembled CM-dextran sensing chip exhibited acceptable repeatability. Before use, the manufactured chip was stored at 4 °C to study the stability of the proposed CM-dextran sensing chip. In this experiment, 1.0 × 10^−8^ g/mL HMGB1 was injected to determine the HMGB1 signal response of different storage periods. The experimental results show that after 21 days of storage, the HMGB1 signal response change was still maintained at 86.0% of the original HMGB1 signal response Figure 12b). This result shows that the proposed CM-dextran sensing chip was stable. The results prove that the proposed CM-dextran sensing chip had good stability and repeatability, and the CM-dextran sensing chip could be prepared without an urgent schedule.

Our results demonstrate the feasibility of constructing a FOLSPR sensor for HMGB1 detection by immobilizing CM-dextran as a bridging molecule on the surface of AuNPs. The FOLSPR sensing technology employs the sensitivity of the noble gold nanoparticle to the refractive index variation in the external environment to detect biological molecules; however, during detections on real samples, it is commonly required to undergo dilution to avoid the change in the refractive index of the external environment. Further, excessive dilution may undesirably degrade the practical detection limits. Since this study mainly verifies the sensing ability of the FOLSPR sensing system combined with CM-dextran, the results show that this method has a lower LOD and a more comprehensive detection range, which can meet the growing number of real sample testing needs. A systematic study is necessary to validate the clinical potential of the FOLSPR for detecting HMGB1 in complex matrices. Therefore, in future clinical applications, we should focus on sample pretreatment and verifying the analytical reliability of actual complex samples, overcome the errors caused by the dilution process, and meet the growing demand for real sample testing.

## 4. Conclusions

This study first proved the innovative method and feasibility of detecting HMGB1 using CM-dextran as a bridging molecule combined with a FOLSPR biosensor. In optimum conditions, anti-HMGB1 was fixed to the sensor and interacted with HMGB1 at various concentrations to realize target detections. These target detections are highly sensitive, selective, repeatable, and reliable. The linear measurement range of the FOLSPR biosensor is 10^−10^ to 10^−6^ g/mL, R^2^ is 0.9928, and LOD is 43.4 pg/mL (1.7 pM). The estimated association rate constant (ka), dissociation rate constant (kd), and affinity constant (kf) of HMGB1 are 2.66 ± 0.2 × 10^5^ M^−1^ s^−1^, 5.71 ± 0.8 × 10^−2^ s^−1^, and 4.66 ± 0.59 × 10^6^ M^−1^, respectively. The RSD values of intra-batch and inter-batch detection of the CM dextran sensing chip are smaller than 2.51% and 2.63%, respectively. In addition, after 21 days of storage of the CM-dextran sensing chip proposed in the storage test, the signal response change was still 86.0% of the initial signal response, showing good stability. As far as we know, this is the first kinetic analysis study on FOLSPR biosensors using CM-dextran as bridging molecules to detect the interaction of HMGB1, and the results are equivalent to that of BIAcore T200. Therefore, this study demonstrated a possible universal method, using CM-dextran as bridging molecules to detect clinical, environmental, and food biomarkers, of which the method has a very high application potential.

## Figures and Tables

**Table 1:** Parameter list of AuNP surface modification biomolecules simulated by DDA.

Gold Sphere Nanoparticle	Diameter (2r_1_)	13.0 nm
	Refractive Index (N_metal_)	Ref [60]
Biomolecular	Radius (r2)	3.2 nm
	Refractive index (n_B_)	1.45
Wavelength	450~800 nm	
Cube size	0.5 nm	
Surrounding medium (n_s_)	1.42	

## Data Availability

Not applicable.
